# Can insulin-like growth factor 1 (IGF-1), IGF-1 receptor connective tissue growth factor and Ki-67 labelling index have a prognostic role in pulmonary carcinoids?

**DOI:** 10.18632/oncotarget.25203

**Published:** 2018-04-27

**Authors:** Georgios A. Kanakis, Lars Grimelius, Dimitrios Papaioannou, Gregory Kaltsas, Apostolos V. Tsolakis

**Affiliations:** ^1^ Department of Pathophysiology, Endocrine Unit, University of Athens Medical School, Athens, Greece; ^2^ Department of Immunology, Genetics and Pathology, Uppsala University, Uppsala, Sweden; ^3^ Department of Pathology, Hygeia Hospital, Athens, Greece; ^4^ Department of Oncology and Pathology, Karolinska Institute, Stockholm, Sweden; ^5^ Cancer Center Karolinska (CCK), Karolinska University Hospital Solna, Stockholm, Sweden; ^6^ Department of Medical Sciences, Uppsala University, Uppsala, Sweden

**Keywords:** pulmonary carcinoids, IGF-1, CTGF, HIF-1, Ki-67

## Abstract

**Introduction:**

Altered expression of Insulin-like Growth Factor-1 (IGF-1), its receptor (IGF-1R), Connective Tissue Growth Factor (CTGF) and Hypoxia Inducible Factor-1 (HIF-1), has been implicated in tumorigenesis. So far, these factors have not been studied systematically in Pulmonary Carcinoids (PCs).

**Aims:**

To examine IGF-1, IGF-1R, CTGF and HIF-1 expression in PCs, and assess their prognostic value over established factors.

**Materials & Methods:**

Retrospective study of 121 PCs (104 Typical and 17 Atypical). The expression of growth factors was studied immunohistochemically and tumors were considered positive if immunoreactivity appeared in >50% of cells.

**Results:**

All studied parameters were expressed in the majority of tumors (IGF-1, IGF-1R, CTGF and HIF-1, in 78.5%, 67%, 72% and 78%, respectively). Their expression tended to be more frequent in TCs and in tumors with Ki-67≤2% (significant only for HIF-1; 82 vs. 53%; p=0.023 and 83 vs. 63%; p=0.025 respectively). CTGF was the only factor correlated with more extensive disease (larger size; presence of lymph node and distant metastases). According to logistic regression analysis, only advanced age, Ki-67≥3.4% and lymph node involvement could predict the development of distant metastases.

**Conclusions:**

IGF-1, IGF-1R, CTGF and HIF-1 are avidly expressed in PCs; however, their presence did not appear to be of statistically significant value over established prognostic factors.

## INTRODUCTION

Pulmonary carcinoids (PCs) are rare tumors of the lungs with neuroendocrine morphology and differentiation. According to the criteria introduced already in the 1990's by Travis et al. [1], they are classified in Typical (TCs) and Atypical Carcinoids (ACs) that exhibit a mostly low and intermediate malignant potential, respectively. However, information regarding the factors involved in the regulation of the biological behavior of PCs is limited. The European Neuroendocrine Tumors Society (ENETS) has suggested a grading system based on the Ki-67 Labelling index (LI) in order to predict the biological behaviour of gastrenteropancreatic neuroendocrine tumors (GEP-NETs) [2]; however, such grading has not been reliably applied in PCs so far, due to the overlapping distribution of the Ki-67 LI between TCs and ACs [3]. As a result, the initial classification scheme proposed by Travis has been reserved until the most recent WHO 2015 Classification [4]. Moreover, no standard guidelines are currently available on medical therapy for PCs due to their poor response to conventional therapeutic regimens [5].There is a need to identify new molecular pathways that may serve as biomarkers to better characterize and classify PCs, as well as potential targets for novel molecular therapies.

Several studies have revealed altered expression of Insulin-like Growth Factor-1 (IGF-1) and its receptor (IGF-1R) in different types of human cancers [6–8], particularly in NETs [9–13]. IGF-1 is a factor essential for cell growth, proliferation and differentiation and preclinical studies in NET cell lines have demonstrated that over-expression of IGF-1 and/or IGF-1R, can induce tumor cell proliferation and raise Chromogranin A (CgA) synthesis [14–16]. So far, data regarding the clinical significance of IGF-1 and IGF-1R expression in GEP-NETs are inconsistent as in some studies their expression has been associated with a more aggressive behavior and poor prognosis [17], whereas in others no such association could be demonstrated [18]. Only a few studies have investigated the expression of IGF-1 and IGF-1R in PCs without generating robust data [11, 12].

Connective Tissue Growth Factor (CTGF) is a member of the cysteine-rich protein 61, connective tissue growth factor and nephroblastoma overexpressed gene (CCN) proteins family that is present in the extracellular matrix and promotes cellular proliferation and migration [19]. Abnormal amplification of CTGF dependent signals also results in excessive tissue repair, leading to scarring and fibrosis [20]. CTGF expression may be triggered by serotonin and/or tissue hypoxia that in turn increases Hypoxia Inducible Factor 1 (HIF-1), a nuclear transcription factor, which interacts with the CTGF promoter [21]. CTGF is expressed abundantly in serotonin producing small intestine carcinoids [22] and in gastric NETs [23]. Moreover, a monoclonal antibody directed against CTGF has been demonstrated to attenuate tumour growth and metastasis in a mouse model of pancreatic cancer [24, 25]. Up to date CTGF expression has not been thoroughly studied in PCs.

The aim of this study is to examine the expression of IGF-1, IGF-1R, CTGF and HIF-1 in PCs by means of immunohistochemistry and assess their prognostic value in comparison to established factors.

## RESULTS

### Patient and tumor characteristics

No significant difference was observed regarding the age at diagnosis and the gender of patients between TCs and ACs (Table 1). The mean tumor size was 20.6 mm (range 2.0 – 70.0 mm) with 93 (77%) tumors exceeding a size of 10mm and 21 (17%) of them exceeding 30mm. ACs were larger than TCs (24.88 vs. 19.83 mm), however this difference was not significant (p=0.226). Patients with ACs presented regional lymph node metastases (LNM), (21% vs. 11.5%, p=0.272) and/or distant metastases more frequently; however, this difference was significant only for the latter (44% vs. 12.0%, p=0.005). In addition, patients with ACs presented more frequently a functioning syndrome (20% vs. 6%), but this difference was also not significant (p=0.104) (Table 1). Urinary 5-HIAA levels were found to be elevated in 4 of 17 patients (23.5%, mean value 321 mg/24hrs); 2 of these patients harboured ACs and 2 TCs. Regarding biological behaviour, the mean Ki-67 LI was significantly higher in ACs than TCs (6.6 ± 4.3% vs. 1.7 ± 2.0%; p<0.001). In the majority of ACs (81.7%) Ki-67 LI was estimated between 3 and 20%, whereas in TCs the majority of tumors (76.5%) had a value ≤2%. No Grade 3 tumors were identified (the highest Ki-67 LI observed was 12%).

**Table 1:** Summary of patients’ demographic and clinicopathological data

	OVERALL	TYPICAL	ATYPICAL	*χ^2^*(t)	p
	(*n*=110)	(*n*=95)	(*n*=15)		
Sex (Female/Male)	66 /44	57/38	9/6	0.000	0.616
Age at diagnosis (years ± SD)	51.97±15.66	52.61±15.07	51.65±18.54	0.203	0.842
Size of primary tumour (mm ± SD)	20.55±12.21	19.83±11.42	24.88±15.94	1.253	0.226
Patients with Localized disease, T_any_N_0_M_0,_ n(%)	69 (73%)	62 (76.5%)	7 (50.0%)	4.231	0.046^*^
Patients with Lymph Node Metastases, T_any_N_1_-_-3_M_any,_ n(%)	19 (14.7%)	15 (11.5%)	4 (21.1%)	1.243	0.272
Patients with Distant Metastases, T_any_N_any_M_1,_ *n* (%)	16 (16.1%)	9 (11.8%)	7 (44%)	10.090	0.005^*^
Ki-67 LI≤2%	89 (73.6%)	85 (81.7%)	4 (23.5%)	χ2 = 25.444	
Ki-67 LI 3-20%	32 (26.4%)	19 (18.3%)	13 (76.5%)	p<0.001^*^	
Patients with functioning tumors, *n* (%)	9 (8.1%)	6 (6.3%)	3 (20.0%)	3.229	0.104

^*^Correlation is significant (2-tailed).

### Controls

Immunostaining with the antibodies against IGF-1, IGF-1R, CTGF and HIF-1, revealed distinct immunoreactive cells in the corresponding control tissues. At the positive controls IGF-1 and IGF-1R immunoreactivity (IR) was strong. Areas of both moderate and strong intensity were identified at the positive controls for CTGF-and HIF-1. No IR was seen after the replacement of the primary antiserum in question by non-immune serum, or after the neutralization test, if available.

### Immunohistochemistry

**Figure 1:**
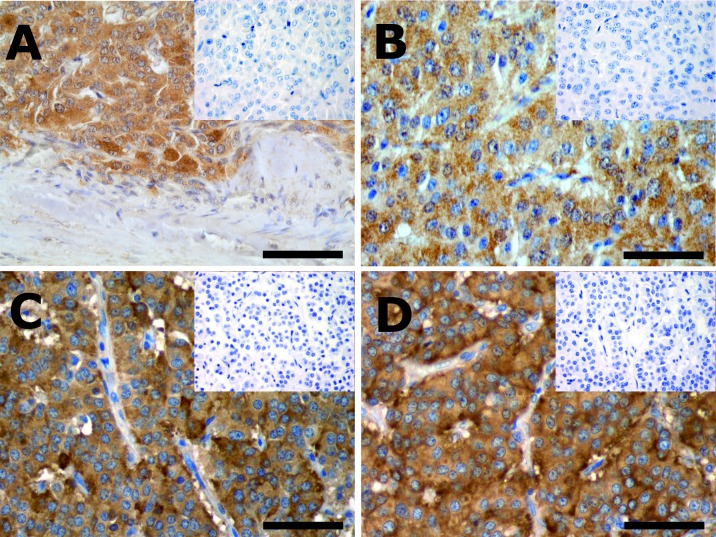
Immunostained sections of a typical (**A and B**) and an atypical (**C and D**) pulmonary carcinoid. (A) shows IGF-1, (B) IGF-1 receptor, (C) CTGF and (D) HIF-1 immunostained tumor sections. Virtually all tumor cells are immunoreactive and the immunoreactivity is cytoplasmic. The tumor capsule (A) and the fibrovascular stroma (B, C and D) are non-immunoreactive and can be used as internal control. Insets represent microphotographs of the neutralization tests (A, B and C) or replacement of the primary antibody by non-immune serum (D). Scale bars= 100 μm.

**Figure 2:**
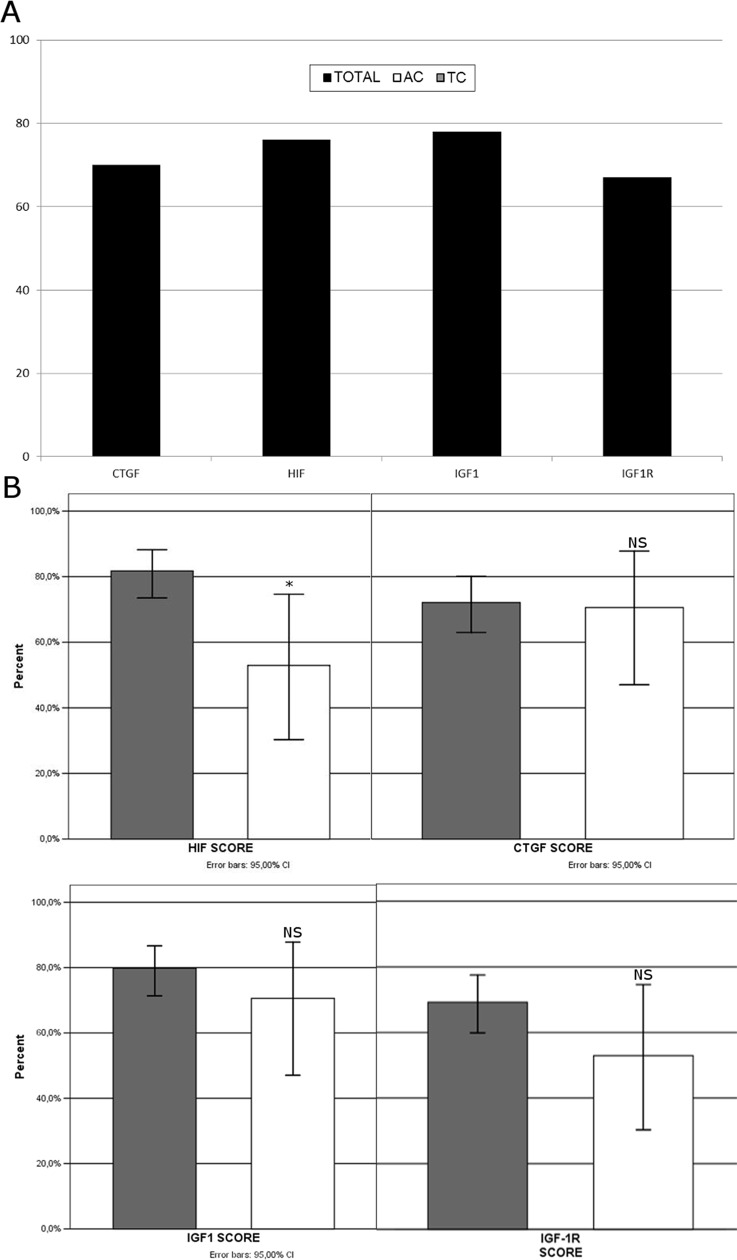
CTGF, HIF-1, IGF-1 and IGF-1R are expressed in the majority of tumors **(A)**. Their expression tended to be more frequent in TCs, although this was significant only for HIF-1 **(B)**.

The vast majority (>90%) of the tumor cells in the studied PCs expressed both CgA and Syn. All studied factors were expressed in the majority of tumors (IGF-1, IGF-1R, CTGF, and HIF-1, in 78.5%, 67%, 72% and 78%, of the cases respectively) and the pattern of immunostaining was exclusively cytoplasmic (Figure 1). At the same tumor specimen the intensity of the IR could vary from negative (0) to strong (3) but only the areas of moderate and strong IR were included as positive. Their expression tended to be more frequent in TCs than in ACs, although this was significant only for HIF-1 (82 vs. 53%, p=0.023; Figure 2). In addition, HIF-1 was expressed more frequently among Grade 1 tumors (83%) vs. Grade 2 tumors (63%, p=0.025), as well as in tumors of small size (<10mm), the latter being not significant. On the contrary, CTGF was predominantly expressed among Grade 2 tumors (not significant), tumors measuring>10mm (79% vs. 50%, p=0.007), tumors accompanied by regional LNM (90% vs. 67%; p=0.056) as well as by distal metastases (89% vs. 65%; p=0.044). The expression of IGF-1 and IGF-1R was not significantly associated with any of the studied parameters, with the exception of IGF-1 being expressed more frequently among non-functioning tumors (81% vs. 50%; p=0.04), a trend also observed in the other studied factors (Table 2). Among the 4 patients who had elevated U-5HIAA all their tumors expressed CTGF; 3 co-expressed IGF-1 with IGF-1R and 2 expressed HIF-1. In three of the patients with increased U-5HIAA, liver metastases were present, associated with high levels of U-5HIAA (mean excretion 640mmol/24h). In the patient without metastases, mean U-5HIAA levels were slightly elevated (72 mmol/24h)

**Table 2:** CTGF, HIF-1, IGF-1 and IGF-1R expression and co-expression in the 121 studied tumors stratified according to Pathological Diagnosis (PAD), grading, tumor size, disease staging and tumor functionality

	HIF(+)	CTGF(+)	IGF-1(+)	IGF-1R(+)	TOTAL n
**PAD**					
**Total**	94 (77.7%)	87 (71.9%)	95 (78.5%)	81 (66.9%)	121
**TC**	85 (81.7%)	75 (72.1%)	83 (79.8%)	72 (69.2%)	104
**AC**	9 (52.9%)	12 (70.6%)	12 (70.6%)	9 (52.9%)	17
**x^2^ (p)**	6.986 (0.023^*^)	0.170 (1.000)	0.736 (0.523)	1.752 (0.265)	
**GRADE**					121
**1**	74 (83.1%)	63 (70.8%)	68 (76.4%)	60 (67.4%)	89
**2**	20 (62.5%)	24 (75.0%)	27 (84.4%)	21 (65.6%)	32
**x^2^ (p)**	5.788 (0.025^*^)	0.206 (0.819)	0.886 (0.455)	0.034 (1.000)	
**Τ**					121
**<=10mm**	23 (82.1%)	14 (50.0%)	21 (75.0%)	20 (71.4%)	28
**>10mm**	71 (76.3%)	73 (78.5%)	74 (79.6%)	61 (65.6%)	93
**x^2^ (p)**	0.417 (0.612)	8.649 (0.007^*^)	0.266 (0.607)	0.331 (0.651)	
**N**	90^†^	83^†^	91^†^	77^†^	^†^117
**0**	75 (77.3%)	65 (67.0%)	76 (78.3%)	62 (64.0%)	97
**1**	15 (75.0%)	18 (90.0%)	15 (75.0%)	15 (75.0%)	20
**x^2^ (p)**	0.050 (0.778)	4.251 (0.056)	0.189 (0.431)	0.610 (0.435)	
**M^‡^**	82^‡^	75^‡^	83^‡^	69^‡^	^‡^109
**0**	71 (78.0%)	59 (64.8%)	70 (76.9%)	60 (65.9%)	91
**1**	11 (61.1%)	16 (88.8%)	13 (72.6%)	9 (50.0%)	18
**x^2^ (p)**	2.306 (0.143)	4.050 (0.044^*^)	0.286 (0.555)	1.030 (0.312)	
**Functioning tumors**					121
**No**	88 (79.3%)	81 (72.9%)	90 (81.1%)	75 (67.6%)	111
**Yes**	6 (60.0%)	6 (60.0%)	5 (50.0%)	6 (60.0%)	10
**x^2^ (p)**	1.967 (0.228)	0.764 0.465)	5.253 (0.037^*^)	0.232 0.433)	

^*^ Correlation is significant (2-tailed);

^†^ data for LNM available for 117 tumors;

^‡^ data for distant metastases available for 109 tumors.

### Statistical analysis

**Figure 3:**
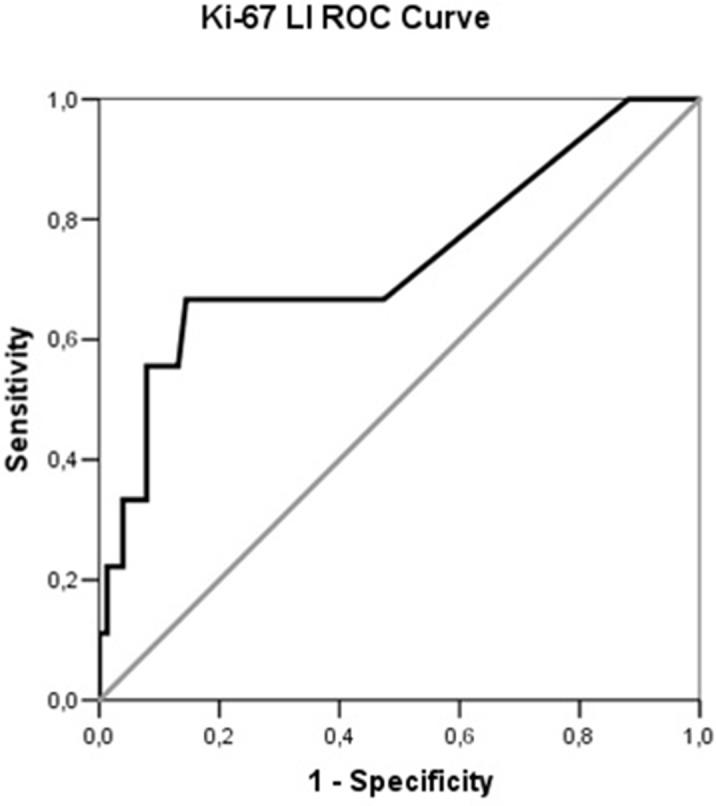
Receiver operating characteristic (ROC) curve illustrating the performance of Ki-67 LI in the prediction of distant metastases in PCs

Considering the presence of distant metastases as a surrogate end-point for adverse disease prognosis, correlations with various clinicopathological parameters based on clinical reasoning and on previous publications, were made (Table 3). A significant positive correlation was demonstrated with the age at diagnosis, the pathological classification (AC vs. TC), Ki-67 LI, grade according to ENETS, the presence of regional LNM, and the presence of a secretory syndrome. In all circumstances the correlation strength was low to moderate, with LNM being the most robust predictor of distant metastases. Regarding the immunohistochemically studied factors; only the presence of CTGF was positively correlated to the presence of distant metastases; however the significance and strength of the correlation were both marginal. The strength of correlation of the classification scheme currently used by WHO (TC vs. AC) was equivalent to the ENETS Grading system (r_s_~0.3). Receiver operating characteristic (ROC) curve was used to define the best cut-off point for Ki-67 LI that could predict the presence of distant metastases. The area under the ROC curve was 0.670 (p=0.023) and a Ki-67 LI value of 3.4% was the optimal cut off with sensitivity 59% and specificity 81% (Figure 3).

**Table 3:** Correlations between the presence of metastatic disease and various clinico-pathological parameters in PCs

	Spearman's rho	Sig. (2-tailed)	N
Gender	0.043	0.696	109
Age at diagnosis	0.263	0.005^*^	109
PAD^a^ (TC vs. AC)	0.304	0.001^*^	109
Ki-67 LI	0.222	0.020^*^	109
Grade	0.322	0.001^*^	109
Tumor Size	0.169	0.080	109
LNM^b^	0.360	<0.001^*^	109
Functionality (+)	0.287	0.003^*^	109
CTGF (+)	0.186	0.053	109
HIF (+)	-0.145	0.131	109
CTGF & HIF(+)	0.054	0.575	109
IGF1 (+)	-0.051	0.597	109
IGF-1R (+)	-0.141	0.143	109
IGF1 & IGF1-R (+)	-0.114	0.239	109

^a^Pathological Diagnosis.

^b^Lymph Node Metastases.

^*^Correlation is significant (2-tailed).

In order to test the effects of these parameters on the likelihood of developing distant metastases, a logistic regression was performed including gender, age at diagnosis, pathological classification (AC vs. TC), grading according to the suggested Ki-67 LI cut off value (3.4%), the size of the primary tumor, the presence of regional LNM, the presence of a secretory syndrome, CTGF, HIF, IGF-1 and IGF-1R expression. The logistic regression model was statistically significant (*χ*^2^ = 27.9, p <0.001), explained 38.9% of the variance Nagelkerke R^2^) and correctly classified 90.5% of cases. However, only age at diagnosis, grading and the presence of regional LNM were significantly associated with an increased likelihood of presenting distant metastases (Table 4).

**Table 4:** Summary of logistic regression analysis for variables predicting the presence of distant metastases in patients with PCs

Variables in the Equation	B		S.E.		Sig.		Exp(B)	95,0% C.I.for EXP(B)	
LNM	2,238		0,700		0,001		9,376	2,376	36,995
GRADE	1,786		0,635		0,005		5,967	1,720	20,706
Age at diagnosis	0,065		0,025		0,010		1,067	1,016	1,120
Constant	-6,611		1,674		0,000		0,001		

Variables not in the Equation	Score	Sig.
Gender	0,075	0,784
PAD (TC vs. AC)	2,108	0,147
Functionality	3,421	0,064
CTGF(+)	3,115	0,078
HIF-1(+)	0,323	0,570
IGF-1(+)	0,788	0,375
IGF-1R(+)	2,953	0,086
Size of Primary	0,000	0,997
Overall Statistics	12,714	0,122

## DISCUSSION

PCs represent the third most frequent type of NETs after rectal (or gastric NETs according to others) and small intestine NETs and a subset may develop metastatic disease; however there is still a lack of robust data regarding their prognosis and management. In the present study avid expression of, IGF-1, IGF-1R CTGF and HIF-1 was documented; however we could not demonstrate a prognostic role, at least superior to established prognostic factors.

Interestingly, the four factors were expressed more frequently in TCs than ACs, though this finding was significant only for HIF-1. In addition, the fact that HIF-1 expression was more frequent in Grade 1 tumors of small size (<10mm), without LN or distant metastases, implies an association of HIF-1 expression with less aggressive tumors. On the contrary, CTGF expression was associated with more aggressive disease as it was predominantly expressed on larger tumors (>10mm) capable of metastasizing to regional LN as well as distant organs. Nevertheless, the correlation of CTGF with advanced disease was weak and did not add significant information in the model that predicted the presence of distant metastases. Finally, a similar trend could not be established regarding IGF-1 and IGF-1R expression.

These apparently equivocal findings add more to the controversy that exists regarding the role of these factors in tumorigenesis. For instance, CTGF over-expression has been positively correlated with aggressive behavior of gastric, pancreatic and breast cancer [26–28], whereas it was shown to inhibit metastasis and invasion of colorectal cancer and lung adenocarcinoma [29, 30]. Regarding NETs, CTGF expression is associated with more advanced disease in ileal and gastric NETs [23, 31]. Similarly, HIF-1 has been recognized in the majority of human cancers as a predictor of poor prognosis [32, 33]; however, such data are not available for NETs. Finally a striking discrepancy exists between preclinical and clinical data regarding the IGF-1 / IGF-1R system in NETs, where despite a strong positive association of these factors with tumorigenesis [34, 35]; relevant anti-IGF-1R therapies have yielded disappointing results [36–38].

The predominant expression of HIF-1 in smaller tumors (<10mm) implies that HIF-1 accumulation is an event that occurs early in tumorigenesis, in contrast to CTGF that was mainly expressed in larger tumors (>10mm). Consequently, it may be assumed that CTGF expression is an epiphenomenon caused by hypoxia and subsequent HIF-1 accummulation when tumor exceeds a critical size. A similar observation for CTGF has also been made in gastric carcinoids, however at a lower cut off point (5.0 mm) [23]. On the other hand, CTGF accumulation represents a critical step in tumor development by triggering other pro-angiogenic factors (such as HIF-1 and VEGF) [39, 40] and preclinical data have shown that CTGF might serve as a promising potential therapeutic target [24, 25].

Recently, much interest has been raised in the treatment of PCs with the mTOR inhibitor everolimus [41]. Preclinical studies suggest that this agent exhibits its antitumor activity through inhibition of IGF-1-dependent pathways [35]. The lack of association of either IGF-1 or IGF-1R with other signs of advanced disease, in combination with the non-promising results of anti-IGF-1 / anti-IGF-1R monotherapy implies that this approach alone is not sufficient to block IGF signalling and that the blockade of additional, downstream IGF-1 pathway components is warranted for the favorable results of everolimus to take effect in PCs.

None of the studied factors added substantial information to the prediction of distant metastases, when they were evaluated in a logistic regression model over established prognostic factors, such as advanced age at diagnosis, the presence of regional LNM and the presence of a secretory syndrome (considering such a syndrome as a result of advanced disease status). Furthermore, we could identify a Ki-67 LI cut off value of 3.4% that was helpful in identifying metastatic from non-metastatic tumors, at least regarding its specificity, and that was also significant in the regression model. This result is in line with others that attempted to apply a grading system for PCs [42]. Reclassification of PCs in two groups according to this cut off (Ki-67 LI <3.4 and ≥3.4) correlated better with the WHO classification scheme than the current ENETS Grading system (r_s_ 0.542 vs. 0.492; P=0.001) and could predict more accurately than both of them the presence of distant metastases (r_s_ 0.322 vs. 0.317 and 0.304 respectively, p=0.001)

In conclusion, this study demonstrated that avid expression of CTGF, HIF-1, IGF-1 and its receptor (IGF-1R) exists in PCs. The detection of these factors predominantly in small, well differentiated tumors implies a role at an early stage of tumorigenesis, apart from CTGF, which was expressed in larger more aggressive tumors. However, logistic regression could not yet prove a prognostic role for these factors, implying that they may represent an auto-regulatory mechanism rather than implicate with the biological behavior of the tumors. A limitation of this study is the small number of tumors included, especially ACs, that may compromise its statistical power. However, PCs are rare tumors and the proportion of included TCs to ACs resembles this observed in the general population. On the other hand, the results regarding the use of K-67 LI were promising and a cut off value of 3.4% could serve as a prognostic factor with sufficient specificity for the likelihood of developing distant metastases.

## MATERIALS AND METHODS

### Patients and tumors

This is a retrospective study of 121 PCs obtained from 110 patients. Some of these tumours have been included in a previous study [43]. Six of the patients harboured multiple pulmonary tumors and two of them tumorlets. In two patients PCs were identified in the context of Multiple Endocrine Neoplasia (MEN) 1. Tumors were classified according to the WHO 2015 criteria [44] in TCs (104 tumors; 86%) and ACs (17 tumors; 14%). In 99 patients staging could be assessed according to available clinical data and patients were grouped to those with disease confined in the lung parenchyma (T_any_N_0_M_0_), disease involving regional LNM (T_any_N_1-3_M_any_) and advanced disease with distant metastases (T_any_N_any_M_1_). Moreover, the rate of cell proliferation was determined by calculating the Ki-67 LI and the tumors were classified in subgroups according to the ENETS Grading classification for GEP NETs (Grade 1: Ki-67 LI≤2%; Grade 2: Ki-67 LI 3-20%); Grade 3: Ki-67 LI>20%) [2] (Table 1).

Nine patients presented with functioning tumors (3 with ectopic ACTH secretion, 4 with typical and 2 with atypical carcinoid syndrome). In 17 patients urinary 5-hydroxyl-indolacetic acid (U-5-HIAA) excretion was measured before treatment initiation, (two 24 hour collections using HPLC and calculated as the mean amount; ULN: 60 mmol/24 h) [45], whereas in one patient with atypical carcinoid syndrome histamine secretion was assessed by measuring urine methylimidazoleacetic acid (UMeImAA), which was found elevated.

### Immunohistochemistry

The neuroendocrine differentiation of the tumors was confirmed by routine immunostaining with the general neuroendocrine markers CgA (mouse monoclonal, LK2H10, Boehringer Mannheim, Germany, dilution 1:2000) and synaptophysin (Syn) (rabbit polyclonal, A0010, Dako Cytomation, Glostrup, Denmark, 1:200) using the Dako Autostainer (Dako, Glostrup, Denmark). Furthermore, the sections were also routinely immunostained with the above system with the Ki-67 antibody (mouse monoclonal, Clone MIB-1, M7240, Dako, Denmark, 1:50).

Immunostaining to detect the expression of IGF-1, IGF-1R, CTGF and HIF-1 was applied using the avidin-biotin complex technique (PK-6100, Vectastain ABC, Vector Laboratories Inc., Burlingame, CA, USA) on tissue sections approx. 4 micrometers thick. The sections were incubated with the antibodies overnight at room temperature. Diaminobenzidine was used as chromogen and Meyer's Haematoxylin for nucleus counterstaining. Before immunostaining, the sections were microwave treated for 2×5 min at 750W using Tris buffer saline, pH 8.0, as retrieval solution.

The primary and corresponding secondary biotinylated antibodies used were as follows:

- Goat polyclonal IGF-1 (C-20), sc-7144, Santa Cruz, (1: 400); Horse anti-goat IgG (H + L) (BA-9500, Vector Laboratories, 1: 100)
- Rabbit polyclonal, IGF-1 Receptor beta (C-20), sc-713 Santa Cruz, (1: 500); Goat anti-rabbit IgG (H + L) (BA 1000, Vector Laboratories, 1: 100)
- Goat polyclonal antibody CTGF L-20, sc-14939, Santa Cruz, (1: 4000); Horse anti-goat IgG (H + L) (BA-9500, Vector Laboratories, 1: 100)
- Mouse monoclonal antibody HIF-1 alpha ESEE 122, NB100-131, Novus Biologicals, (1: 3000); Horse anti-mouse IgG (H + L) (BA-2000, Vector Laboratories, 1: 100)

### Controls

Tissue from normal liver and kidney were used as positive controls for IGF-1 and IGF-1R respectively, whereas specimens from ileal carcinoid and neuroblastoma served as positive controls for CTGF and HIF-1 respectively. “Negative” immunostaining controls were generated after replacement of the primary antibody by non-immune serum at the same dilution as the primary antibody in question and in the same diluent. In occasional cases, a neutralization test was conducted following a 24-hour incubation of the primary antiserum with the relevant antigen (10nmol antigen per ml diluted antibody solution) before application to the sections. The peptides used for the neutralization test were:

- IGF-1 (C-20P), sc-7144P, Santa Cruz
- IGF-1 Receptor beta (C-20P), sc-713P Santa Cruz
- CTGF (L-20P), sc-14939P, Santa Cruz

Neutralization test was not performed for HIF-1 since a relevant peptide was not available.

### Intensity score

The intensity of the immunostaining was divided as 0 for negative, 1 for weak IR, 2 for moderate, and 3 for strong. Tissue areas with only moderate and strong IR were regarded as positive.

### Calculation of the relative incidence of immunoreactive cells

In consecutive immunostained sections, the relative incidence of immunoreactive cells in relation to total tumor area was estimated by light-microscopy at a magnification of X400 using a square grid in one of the oculars. At least five randomly selected areas were examined; in smaller lesions the whole of the neoplastic tissue. Tumors were considered as positive if IR appeared in the majority (>50%) of the tumor cells. The Ki-67 LI was calculated by counting the immunoreactive tumor cells at a magnification of X400. To facilitate the counting, a square grid was placed in one of the oculars. At least 2000 tumor cells were counted in the most proliferative tumor areas. The labelling index was expressed as the percentage of Ki-67 immunoreactive cells.

Mitotic activity was estimated in Haematoxylin and Eosin (H&E) stained tissue sections, analysing 50 High Power Fields, (HPFs) and the mitotic index was expressed as the number of mitoses/10 HPFs.

The calculation of Ki-67 and mitoses was performed by one of the authors (LG). The remaining immunohistochemical analysis was done by two investigators (L.G. and A.V.T.) in consensus.

### Statistical analysis

Tumors were stratified according to their size (largest dimension) and two cut off values were selected; 30mm and 10mm. The first cut off point was selected according to the TNM classification system proposed by the American Joint Committee on Cancer, as it is the limit between T1 and T2 tumors (AJCC cancer staging manual 7^th^ Ed. 2010), whereas the latter was demonstrated in a previous study to be associated with increased expression of CTGF [23]. For comparisons between nominal and quantitative data Fisher's exact and Student's *t* test was used respectively, whereas correlations between immunohistochemical findings and clinico-pathological parameters were tested using Spearman's rank correlation coefficient. Receiver Operating Characteristic (ROC) curve was used to assess the performance of the employed parameters. A forward stepwise logistic regression was performed to test the effects of different parameters as prognostic factors for the development of distant metastases. The level of statistical significance was set to 0.05. Calculations were performed using Statistical Package for Social Sciences (SPSS, Inc., Chicago, IL, USA) V.13.0 software.
